# Prevention of diabetic ketoacidosis at diagnosis of type 1 diabetes: Are certified athletic trainers an untapped resource?

**DOI:** 10.1002/edm2.188

**Published:** 2020-09-28

**Authors:** Kaleb T. Bogale, Tamara K. Oser, Alexander Zettlemoyer, Jessica Parascando, Matthew L. Silvis

**Affiliations:** ^1^ Penn State College of Medicine Hershey PA USA; ^2^ Department of Family and Community Medicine Penn State College of Medicine Hershey PA USA; ^3^ Department of Family Medicine University of Colorado Anschutz School of Medicine Aurora CO USA; ^4^ Athletic Medical Services at Mechanicsburg Area Senior High School Mechanicsburg PA USA; ^5^ Department of Orthopedics and Rehabilitation Penn State College of Medicine Hershey PA USA

**Keywords:** certified athletic trainer, diabetes mellitus, diabetic ketoacidosis, type 1

## Abstract

**Aims:**

To assess the knowledge of certified athletic trainers (ATs) on the presenting signs and symptoms of type 1 diabetes (T1D).

**Methods:**

We conducted a 31‐question survey of secondary school ATs recruited from the National Athletic Training Association that established demographic information, knowledge of presenting signs and symptoms of T1D, and previous personal or professional exposure to individuals with T1D. We report descriptive statistics and univariate analyses evaluating the characteristics associated with T1D knowledge. We then report a multivariable model incorporating age, gender, years of experience and education level with T1D knowledge as the dependent variable.

**Results:**

128 participants (92f:34m) met inclusion criteria and were included in this study. The majority of participants correctly identified frequent thirst (96.1%, *n* = 123) and frequent urination (85.9%, *n* = 110) as common presenting signs and symptoms of T1D, while fewer participants identified weight gain (58.6%, *n* = 75) or joint pain (39.1%, *n* = 50) as incorrect presenting signs and symptoms of T1D. Participants with over ten years of experience or previous exposure to individuals with T1D had increased T1D knowledge. Participants with advanced education (Master's degree or Doctorate) had no statistically significant difference in T1D knowledge compared to those with a Bachelor's degree. The only factor that demonstrated a significant association with T1D knowledge on multivariable analysis was the female gender.

**Conclusions:**

Educational awareness campaigns of T1D symptoms to reduce the rate of DKA at diagnosis of T1D have never included ATs. This study illustrates the importance of targeting future educational interventions on newly trained ATs.

## Graphical abstract

Educational interventions on presenting symptoms of type 1 diabetes (T1D) to childhood contacts have successfully reduced the rate of diabetic ketoacidosis at diagnosis of T1D. No previous educational interventions have included athletic trainers (ATs) and no literature reported ATs knowledge of the presenting symptoms of T1D and characteristics associated with increased knowledge. We report experienced ATs (>10 years) or previous exposure to an individual with T1D was positively associated with T1D knowledge in univariate analysis, while the only factor that demonstrated an association in multivariable analysis was the female gender.

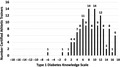

- Educational interventions on the common presenting symptoms of type 1 diabetes (T1D) to childhood contacts have successfully reduced the rate of diabetic ketoacidosis at diagnosis of T1D.
- No previous educational interventions have included athletic trainers (ATs). No literature reported ATs knowledge of the presenting symptoms of T1D and characteristics associated with increased knowledge.
- Experienced ATs (>10 years) or previous exposure to an individual with T1D was positively associated with T1D knowledge in univariate analysis. The only factor that demonstrated an association in multivariable analysis was the female gender. Our study highlights the importance of targeting newly trained ATs in future T1D education.

## 1 INTRODUCTION

The overall incidence rate of type 1 diabetes (T1D) in United States youth has increased 1.9% each year from 2001 to 2015, with the greatest incidence rate being in children aged 10‐14 and 15‐19.^1^ The majority of children with new‐onset T1D present with one or two of the four main symptoms of diabetes: polyuria, polydipsia, weight loss and tiredness.^2^ It has been reported in the United States that approximately 30% of new‐onset T1D cases progressed to diabetic ketoacidosis (DKA) before diagnosis.^3^ Presentation during DKA has devastating complications including increased morbidity, mortality and psychological distress, and is a predictor of poor long‐term glycaemic control.^4^ In addition, those diagnosed during DKA have a significantly higher use of healthcare services and increased medical costs.^5^ Lack of awareness by patients and physicians of the presenting signs and symptoms of diabetes accounts for the majority of DKA cases at time of T1D diagnosis.^6^

Attempts to improve public awareness of the presenting signs and symptoms of T1D have been successful in decreasing rates of DKA during diagnosis of T1D.^7^ In the United States, these T1D awareness campaigns have focused on patients, parents, school teachers and healthcare providers.^8^ Certified athletic trainers (ATs) often spend considerable time with student athletes,therefore, we hypothesize that it is especially important that they are targeted in future T1D awareness campaigns.^9^ ATs, as a member of the healthcare team, should identify and refer athletes presenting with signs and symptoms of T1D.^10^ There is a gap in the literature regarding ATs ability to identify presenting signs and symptoms of T1D and factors, including educational background, that influence AT knowledge of T1D.

This study aimed to bridge that gap by assessing the knowledge of presenting signs and symptoms of T1D and identifying factors that influence this knowledge through a national survey of ATs in the United States.

## 2 PARTICIPANTS

A national survey was powered to include at least 125 participants to capture the participants estimated to obtain statistical significance for differences in AT knowledge of T1D presenting symptoms. The survey was distributed electronically to 2975 secondary school ATs at a cost of $0.15 per survey, thus collecting data beyond what was statistically necessary was beyond the scope of the budget. The survey was subsequently closed after reaching the desired sample size. The survey was distributed in accordance with the National Athletic Training Association internal process to obtain a broad representative sample of ATs throughout a three‐week span in October 2019. Inclusion criteria included ATs in the United States, able to read, write and communicate in English, have Internet access, and willing to consent and complete study requirements.

## 3 METHODS

The cross‐sectional survey study was approved by the Institutional Review Board at Penn State College of Medicine. Before proceeding to the survey, participants read a summary of explanation of research and were asked if they agreed to participate in the study. They were also asked eligibility questions, which either allowed them to continue to the survey, or notified them that they were not eligible, and thanked them for their time.

A 31‐question survey was constructed using Qualtrics survey generation software.^11^ The survey featured questions on the presenting signs and symptoms of T1D (T1D knowledge assessment), certified athletic trainer‐specific information, and demographics. The T1D knowledge assessment gave participants a clinical presentation that included either a true symptom of T1D (frequent thirst, frequent urination, changes in behaviour/mood and changes in appetite) or a false symptom of T1D (joint pain and weight gain). They were given three answer options: true, false or unsure. They were also asked to rate their confidence level on their answer choice (very confident, somewhat confident, or not confident). Certified athletic trainer‐specific information included professional and personal encounters with individuals living with T1D, frequency ATs reported undiagnosed T1D in an athlete, and ATs opinion on continuing education in type 1 diabetes. Demographic information included age, sex, zip code, race and education level.

### 3.1 Statistical analysis

**FIGURE 1:**
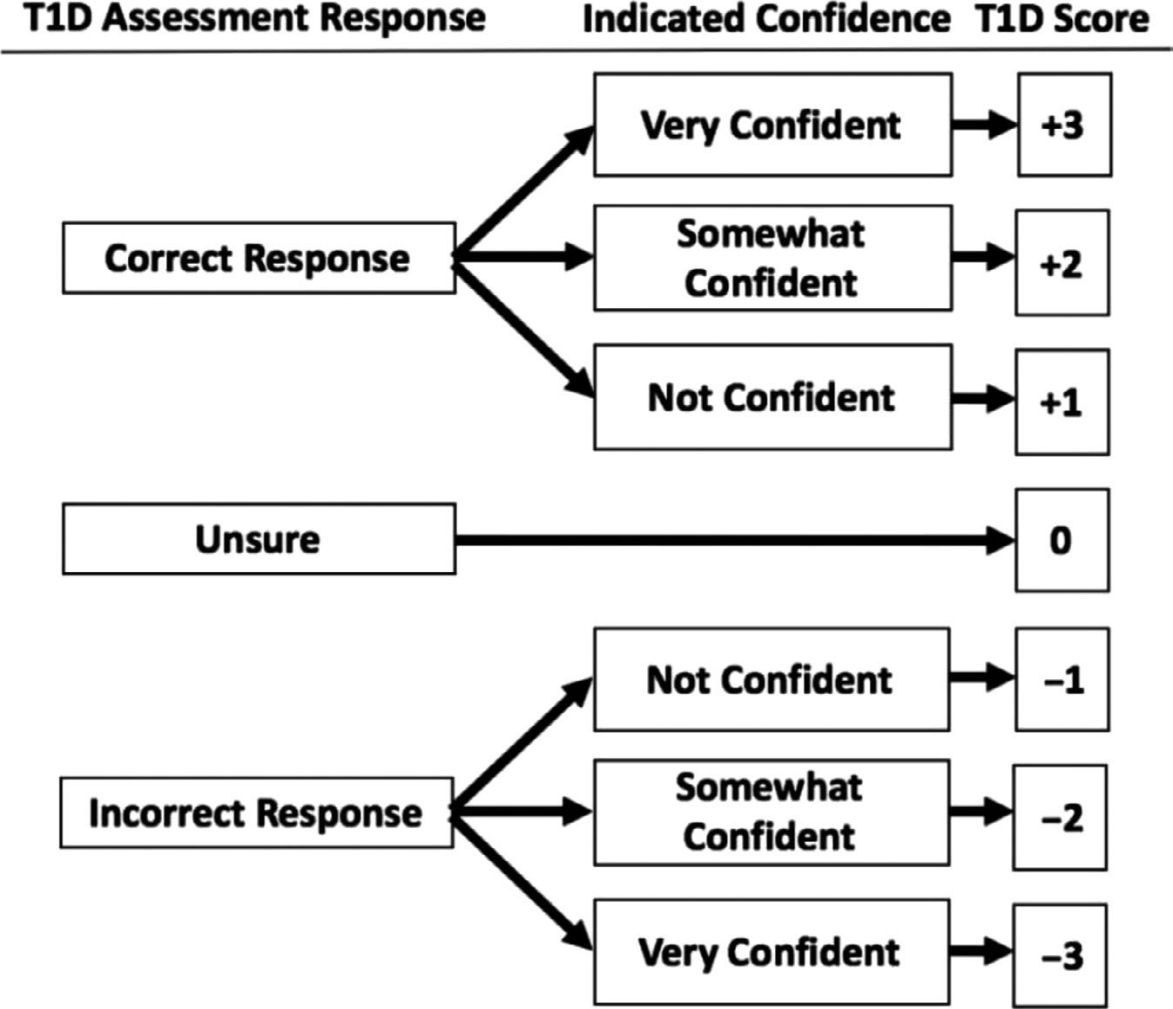
The scoring criteria for each question on the presenting signs and symptoms of type 1 diabetes (T1D) assessment incorporates confidence level. The range of the six‐question T1D knowledge survey was −18 to 18

The T1D knowledge assessment (range: −18 to 18) was scored based on the ability to correctly identify true and false symptoms of T1D. The scoring incorporated the ATs confidence level, as shown in Figure 1. ATs were dichotomized into two groups (low or high knowledge) based on their T1D knowledge score in relation to the median score.

First, differences in T1D knowledge were examined using two‐sample t tests for demographics (race, gender and education level), professional and personal encounters with individuals living with T1D, and years of experience as an AT. An analysis of variance test determined the temporal impact of years of experience on T1D knowledge. We then report a multivariable model incorporating age, gender, years of experience and education level with T1D knowledge as the dependent variable.

## 4 RESULTS

A total of 128 participants (response rate = 6.4%) completed the survey, including representation from 35 states. Demographic information is presented in Table 1. The participants were primarily female (71.9%, *n* = 92), aged 25‐34 years (46.1%, *n* = 59), white (93.8%, *n* = 120), worked in a suburban community (58.6%, *n* = 75), and had a Master's (66.4%, *n* = 85) or Doctorate (3.1%, *n* = 4) degree in athletic training. Most participants have encountered an athlete living with T1D in a professional setting (87.2%, *n* = 109) or had personal exposure to an individual living with T1D (73.2%, *n* = 93). The majority of participants did not feel adequately prepared for the management of an athlete living with T1D (64.5%, *n* = 80) and are interested in continuing education in presenting signs and symptoms of T1D (93.4%, *n* = 114).

**TABLE 1:** Baseline characteristics of certified athletic trainers’ impact on type 1 diabetes knowledge

Variable, *n* (%)	High T1D knowledge (*n* = 60)	Low T1D knowledge (*n* = 68)	*P*‐value
Age			
<24	1 (1.7%)	12 (17.6%)	**.005**
25‐34	24 (40.0%)	35 (51.5%)	
35‐44	14 (23.3%)	7 (10.3%)	
45‐54	14 (23.3%)	10 (14.7%)	
>54	7 (11.7%)	4 (5.9%)	
Sex			
Female	43 (71.7%)	49 (72.1%)	.96
Male	17 (28.3%)	17 (25.0%)	
Other	0 (0.0%)	1 (1.5%)	
Prefer not to answer	0 (0.0%)	1 (1.5%)	
Race			
White	59 (98.3%)	61 (89.7%)	.49
American Indian or Alaska Native	1 (1.7%)	1 (1.5%)	
Asian	0 (0.0%)	1 (1.5%)	
Black or African American	0 (0.0%)	1 (1.5%)	
Native Hawaiian or other Pacific Islander	0 (0.0%)	1 (1.5%)	
Other	0 (0.0%)	3 (4.3%)	
Ethnicity			
Hispanic or Latino	2 (3.3%)	7 (10.3%)	.17
Not Hispanic or Latino	58 (96.7%)	61 (89.7%)	
Setting			
Rural	16 (26.75%)	11(16.2%)	.35
Suburban	33 (55.0%)	42 (61.8%)	
Urban	11 (18.3%)	15 (22.1%)	
Education			
Bachelor's Degree	21 (35.0%)	16 (23.5%)	.17
Master's Degree	36 (60.0%)	49 (72.1%)	
Doctorate of Athletic Training	1 (1.7%)	3 (4.4%)	
Other	2 (3.3%)	0 (0.0%)	
Years of experience			
0‐5	10 (17.9%)	27 (40.9%)	**.003**
6‐10	9 (16.1%)	18 (27.3%)	
11‐15	9 (16.1%)	4 (6.1%)	
>15	28 (50.0%)	17 (25.8%)	

Bolded values represent statistical significance demonstrated with a *P*‐value < .05.

**FIGURE 2:**
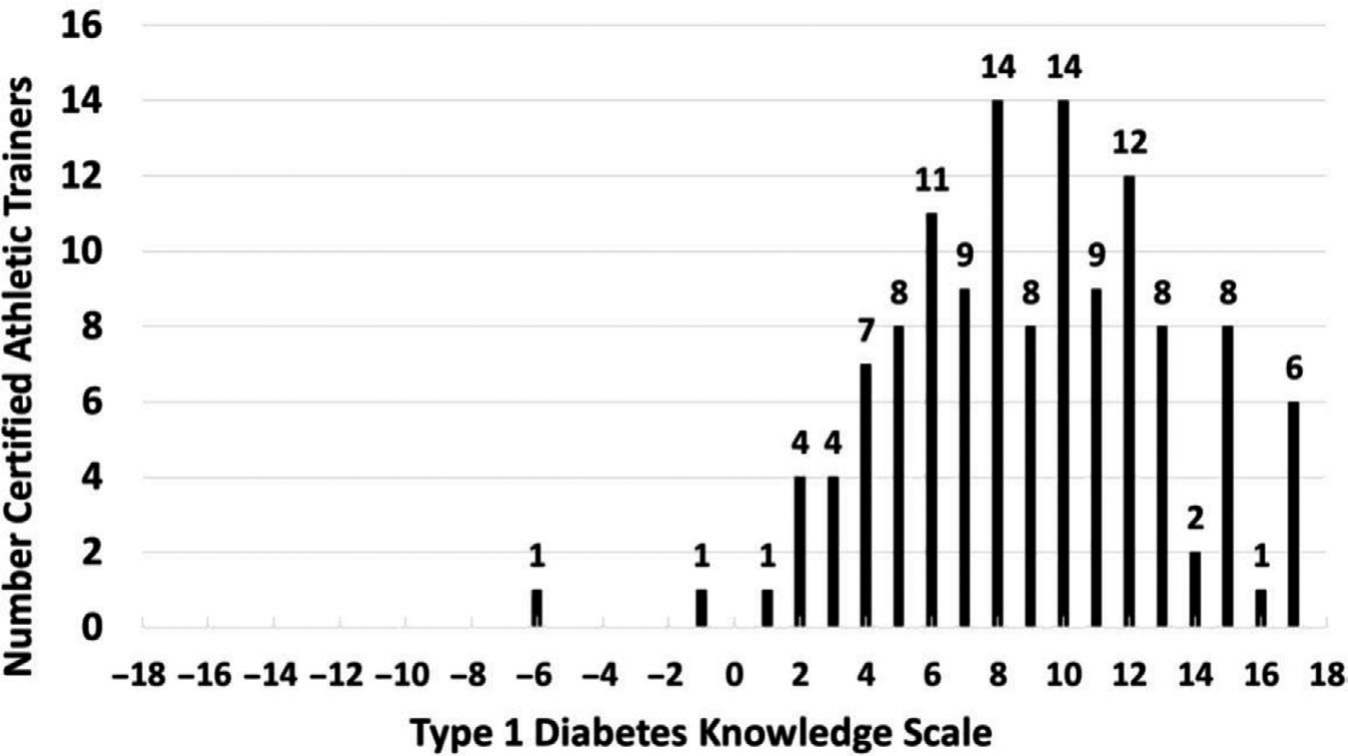
Distribution of 128 certified athletic trainer (ATs) scores on type 1 diabetes (T1D) knowledge assessment (median = 9). ATs were separated into either (1) high T1D knowledge (*n* = 60) or (2) low diabetes knowledge (*n* = 68) based on their T1D knowledge score relative to the median score

The majority of participants identified common presenting signs and symptoms of T1D such as frequent thirst (96.1%, *n* = 123), frequent urination (85.9%, *n* = 110), changes in behaviour and mood (80.5%, *n* = 103) and changes in appetite (75.8%, *n* = 97). Fewer participants identified weight gain (58.6%, *n* = 75) or joint pain (39.1%, *n* = 50) as incorrect signs and symptoms of T1D. Figure 2 shows the distribution of participant scores on the T1D knowledge assessment (median = 9). Participants were assigned to a high (*n* = 60) or low (*n* = 68) T1D knowledge group based on their T1D knowledge score relative to the median score.

An increased age (*P* = .005) and years of experience as an AT (*P* = .003) were positively associated with T1D knowledge, particularly among ATs with 11‐15 years (*P* = .02) or greater than 15 years (*P* = .001) experience. Both previous professional (*P* = .04) and personal (*P* = .02) exposure to an individual with T1D was positively associated with T1D knowledge. Participants with advanced education (Master's degree or Doctorate in athletic training) had no significant difference in T1D knowledge compared to those with a Bachelor's degree in both univariable and multivariable analysis. There were no statistically significant differences in T1D knowledge based on race or area of work (rural, suburban or urban). The only factor that demonstrated a significant association with T1D knowledge on multivariable analysis was the female gender (*P* = .03). While the majority of participants (71.6%, *n* = 91) never raised concern of T1D in an undiagnosed athlete, those with increased T1D knowledge were more likely to have reported their concern of undiagnosed T1D in an athlete (*P* = .03).

## 5 DISCUSSION

In efforts to improve early detection of T1D, there have been attempts to educate child contact points on the presenting signs and symptoms of T1D (polyuria, polydipsia, weight loss and tiredness).^2^, ^3^ The early identification of these symptoms may prevent the development of DKA, improve health outcomes, decrease financial costs and reduce psychological distress associated with DKA.^4^, ^5^ ATs are part of a circle of contact points for children and likely to spend more time with student athletes with diabetes than a physician and, therefore, could be an important part of efforts to diagnose T1D prior to the development of DKA.^9^, ^10^ To the best of our knowledge, there are no prior studies examining ATs knowledge of the presenting signs and symptoms of T1D and factors associated with increased T1D knowledge.

Our first research objective is the initial determination of secondary school ATs knowledge of the presenting signs and symptoms of T1D. This study specifically focused on secondary school ATs because the incidence rate of T1D is highest among children ages 10‐14 and 15‐19.^1^ In our study, the majority of ATs correctly identified frequent thirst (96.1%, *n* = 123) and frequent urination (85.9%, *n* = 110) as common presenting signs and symptoms of T1D. There were fewer ATs able to identify weight gain (58.6%, *n* = 75) as an incorrect symptom of T1D. Knowledge of the four main presenting symptoms of T1D (polyuria, polydipsia, weight loss and fatigue) is critical for early diagnosis of T1D because all children with new‐onset T1D present with one, and 98% present with two of the four main symptoms.^2^ Future education of ATs should emphasize these four recognizable symptoms because our data support ATs with increased T1D knowledge were more likely to have reported their concern of undiagnosed T1D in an athlete.

Our second research objective is identifying factors that influence ATs knowledge of T1D. We are the first to report that ATs with a Bachelor's degree had no significant difference in T1D knowledge than ATs with advanced education of a Master's degree or Doctorate in athletic training. We hypothesize this may result from limited requirements in post‐professional education for the identification and referral of T1D. Currently, undergraduate AT education consists of basic identification of T1D signs and symptoms for referral to the athlete's primary care provider or qualified members of the sports medicine team for disease confirmation. Following the diagnosis of T1D, ATs continue to monitor the athletes’ signs and symptoms and facilitate adherence to the athletes’ prescribed diabetic treatment plan. Management includes care of emergent and nonemergent hyperglycaemia and hypoglycaemia.^10^

Beginning in 2022, The Commission on Accreditation of Athletic Training Education requires professional athletic training programs to be at the Master's degree level to maintain accreditation.^12^Our data do not support an increase in T1D knowledge with increased educational requirements. It is well known that early identification of T1D in athletes can optimize patient outcomes.,^1^ ^,^ ^34^ As a frontline member of the sports medicine team and perhaps the only member of the team seen daily by the athlete, it is imperative that ATs have the education and tools to identify and refer suspected undiagnosed T1D in athletes.^10^

Our data suggest ATs are learning from their on‐the‐job experience managing an athlete with T1D. Participants with over ten years of experience and previous exposure to individuals with T1D had increased T1D knowledge. These findings are consistent with a 2007 survey of 193 ATs, which identified AT age and years of experience as predictors of increased general diabetes knowledge.^13^ Our findings highlight the importance of focusing future education interventions on newly trained ATs. Utilizing the shift in 2022 to a Master's degree graduation requirement is an opportunity to improve post‐graduate AT education of presenting signs and symptoms of T1D. Future studies can identify any subsequent impact on the frequency and accuracy of AT identification of undiagnosed T1D in athletes after an educational intervention. In conclusion, we recommend the integration of ATs into future public awareness campaigns on presenting signs and symptoms of T1D as a potential inexpensive solution to reduce rates of DKA among children.

## CONFLICTS OF INTEREST

Dr Tamara Oser ‐Consultant, Cecilia Health (current). Consultant and grantee, Helmsley Charitable Trust (current). Consultant, Glucagon Multidisciplinary Working Group, Xeris Pharmaceuticals (2019). Primary Care Consultant, Afrezza Inhaled Insulin Working Group, MannKind Corporation (2019). No other authors report any conflicts of interest.

## AUTHOR CONTRIBUTIONS

KTB, TO, AZ, JP and MS performed and designed the research study and wrote and edited the final manuscript. KTB and JP performed the data analysis.

## Data Availability

The data that support the findings of this study are available from the corresponding author upon reasonable request.
